# Prenatal Diagnosis of Down Syndrome Associated with Right Aortic Arch and Dilated Septum Cavi Pellucidi

**DOI:** 10.1155/2012/329547

**Published:** 2012-09-27

**Authors:** José Morales-Roselló, Rafael Lázaro-Santander

**Affiliations:** ^1^Servicio de Obstetricia y Ginecología, Hospital de la Plana, 12540 Villarreal, Spain; ^2^Servicio de Anatomía Patológica, Hospital de la Plana, 12540 Villarreal, Spain

## Abstract

A 30-year-old woman with a normal first trimester Down syndrome screening attended our ultrasound unit for a 20-week scan. The most remarkable anomalies were the presence of a right aortic arch along with a dilated cavum septi pellucidi. In addition, the scan showed an atrioventricular canal and bilateral choroid plexus cysts. Fetal karyotype showed the existence of trisomy 21. A novel association between Down syndrome and dilated cavum septi pellucidi is reported and the relationship between DS and vascular rings is discussed.

## 1. Introduction

Despite both conditions present retroesophageal rings amenable to prenatal diagnosis, Down syndrome (DS) has been associated with left aortic arch plus aberrant right subclavian artery (LAA/ARSA), but not with right aortic arch plus left subclavian artery (RAA/ALSA). We present a fetus with this latter association together with a dilated cavum septi pellucidi, an anomaly never described in association with DS.

## 2. Case Presentation

**Figure 1:**
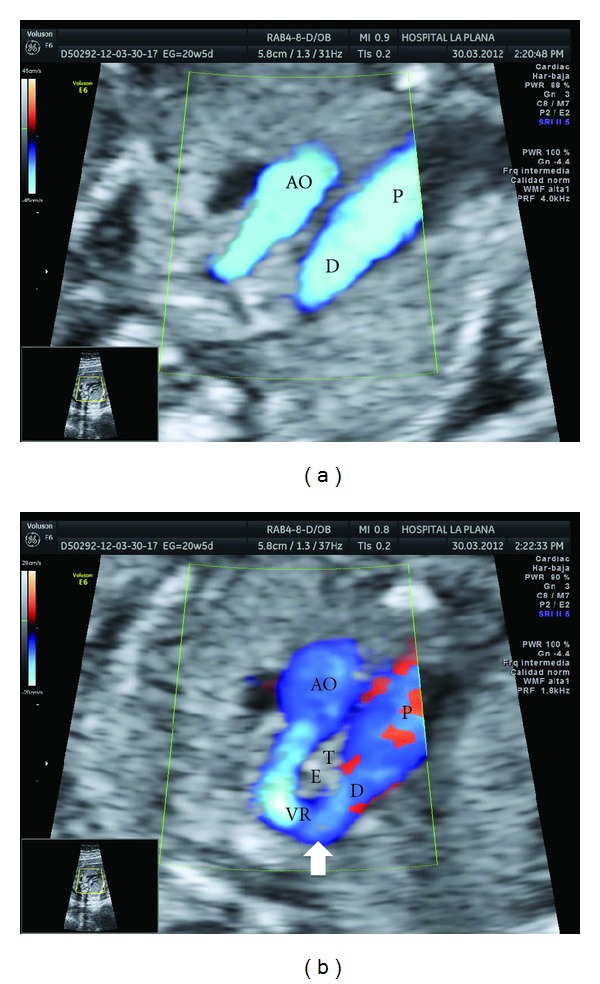
(a) Color Doppler ultrasound (three vessels and trachea view) showing two parallel vessels. This was the first clue to the diagnosis of cardiac malformation and subsequent fetal aneuploidy. AO: aortic arch, P: pulmonary vein, D: ductus arteriosus. (b) Color Doppler ultrasound showing a vascular retroesophageal ring formed with the union of the right aortic arch and the prolongation of the ductus arteriosus. As the aortic arch passes over the right bronchus, the encounter between both circulations occurs behind the esophagus. AO: aortic arch, P: pulmonary vein, D: ductus arteriosus, VR: vascular ring, T: trachea, E: esophagus. In the union of the ductus arteriosus and the aortic arch a small outpouching is seen forming a small diverticulum of Kommerell (arrow), where the aberrant left subclavian artery (not seen in this picture) joined.

**Figure 2:**
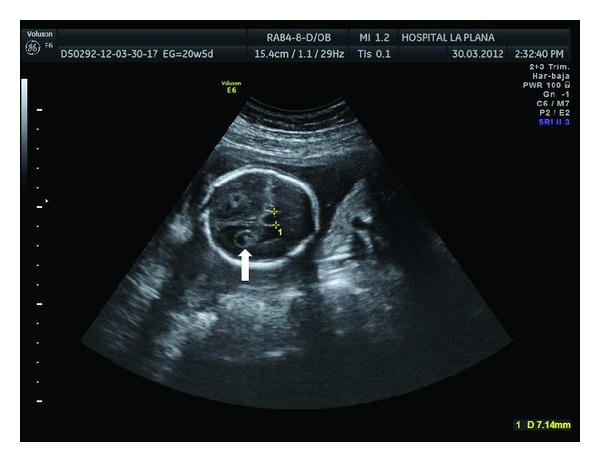
Dilated cavum septi pellucidi (7.14 mm) and bilateral choroid plexus cysts (arrow).

**Figure 3:**
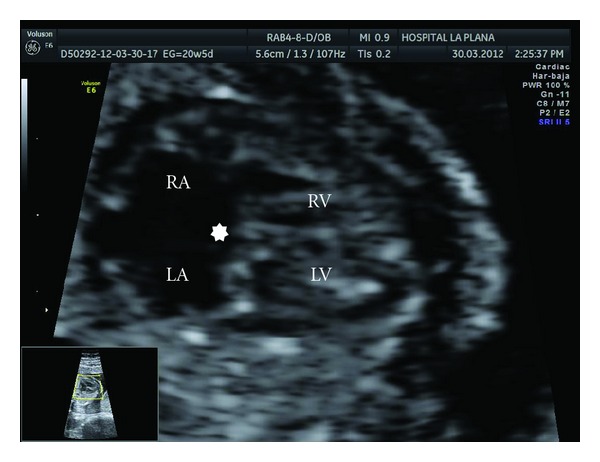
Atrioventricular canal. Note the absence of the crux cordis (star). RA: right atrium, LA: left atrium, RV: right ventricle, LV: left ventricle.

A 30-year-old woman in her second pregnancy, with no remarkable past medical history, and a normal first trimester Down syndrome screening (including free-*β*-HCG/PAPP-A, nuchal translucency, nasal bone, and ductus venosus flow), attended our unit for the 20 weeks scan. The most remarkable anomalies were the presence of two parallel vessels on the three vessels and trachea view, ending in a retroesophageal vascular ring (Figures 1(a) and 1(b)), plus a dilated cavum septum pellucidum, and bilateral choroid plexus cysts (Figure 2). A detailed scan also showed a bulbous structure in the union of the ductus arteriosus and the aortic arch resembling a small diverticulum of Kommerel and the presence of an atrioventricular canal (Figure 3) which raised the suspicion of trisomy-21. An amniocentesis was performed and a fetal FISH and karyotype was requested to rule out aneuploidies and microdelections 22q11.2 with the result of trisomy-21 and a normal chromosome 22.

**Figure 4:**
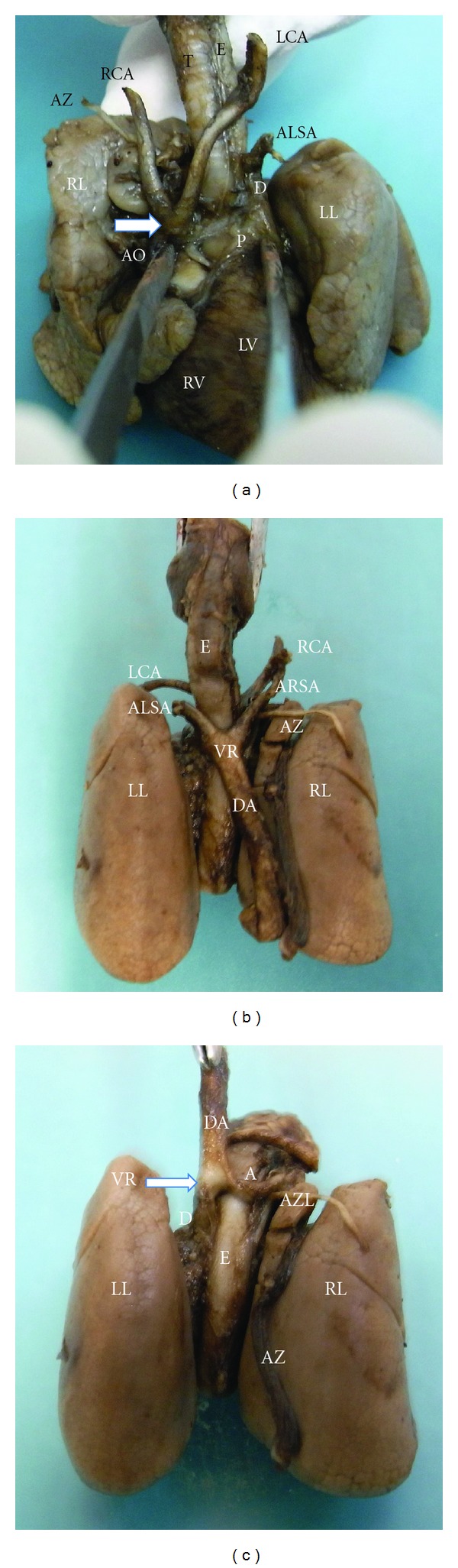
(a) Anterior view of the mediastinum. The pulmonary artery (P) and ductus arteriosus (D) are seen crossing over the left bronchus towards a right-sided descending aorta. An aberrant left subclavian artery (ALSA) is seen branching at the union between the ductus and the aorta. Both left and right carotid arteries (RCA/LCA) branch from the aortic arch (AO) anteriorly to the trachea in a V-shape set up. We could not confirm the presence of a diverticulum of Kommerel at the origin of the left subclavian artery. An aberrant azygos vein (AZ) is seen separating the upper lobe of the right lung forming an azygos lobe. RL: right lung, LL: left lung, T: trachea, E: esophagus. (b) Posterior view of the mediastinum. Aberrant left and right subclavian arteries (ALSA/ARSA) are seen branching from the vascular ring (VR) in the union between the ductus and the aorta. Both left and right carotid arteries (LCA/RCA) are seen coming from the anterior mediastinum after branching from the aortic arch. An aberrant azygos vein (AZ) is seen separating the upper lobe of the right lung forming an azygos lobe. RL: right lung, LL: left lung, E: esophagus, DA: descending aorta. (c) Posterior view of the mediastinum. The descending aorta has been moved upwards in order to see the vascular ring (VR) (arrow). The ductus arteriosus (D) is seen crossing over the left bronchus joining the descending aorta (DA). A right aortic arch (A) is seen surrounding the esophagus (E). An aberrant azygos vein (AZ) was seen separating the upper lobe of the right lung forming an azygos lobe (AZL). RL: right lung, LL: left lung, E: esophagus.

After termination of pregnancy, the postmortem study confirmed the sonographic findings (Figures 4(a), 4(b), and 4(c)) showing a retroesophageal ring formed with the union of the aortic arch and the ductus arteriosus, which ended in a descending right-sided aorta. The aortic arch passed over the right bronchus and joined the ductus arteriosus behind the esophagus. Both carotid arteries branched from this aortic arch anteriorly to the trachea in a V-shape set up.

In addition, two aberrant right and left subclavian arteries branched from the retroesophageal vascular ring, although the presence of a diverticulum of Kommerel at the origin of the left subclavian artery could not be confirmed. Finally, an aberrant azygos vein was seen separating the upper lobe of the right lung forming an azygos lobe.

## 3. Discussion

Down syndrome (DS) is usually detected at 12 weeks with the combined DS screening or at 20 week with secondary markers and associated malformations [1]. In this fetus, however the screening was normal and the most significant findings at 20 weeks were the presence of a complete vascular ring and a dilated cavum septi pellucidi, both conditions unrelated with DS. 

Vascular rings diagnosed prenatally correspond either to double aortic archs (DAA) (with an “O” shape), left aortic archs plus aberrant right subclavian artery (LAA/ARSA) (with an “V” shape), or right aortic archs with aberrant left subclavian artery (RAA/ALSA) (with an “U” shape) [2]. Complete vascular rings in the 3VT view are usually caused by RAA/ALSA or DAA, whereas incomplete vascular rings are usually caused by LAA/ARSA [3]. Vascular rings are frequently diagnosed after birth in patients with dysphagia or cough [4], however prenatal diagnosis at 20 weeks may be also achieved using the three-vessel and trachea (3VT) view proposed by Yagel et al. [5].

DS has been associated with the existence of a LAA/ARSA. In fact, the presence of an ARSA has been recently proposed as an independent marker of DS [6]. Conversely, few reports have associated DS with the RAA/ALSA [2, 7]. Therefore, despite half of prenatally diagnosed vascular rings correspond to RAA/ALSA, only few are chromosomically abnormal. To increase the rarity of this case, the fetus presented a dilated cavum septi pellucidi, a rare malformation [8] undescribed in relation with DS. In this fetus, the cavum was two times the normal measurement, exceeding by far the 2SD limit [9]. 

The scan also showed the presence of bilateral choroid plexus cysts and an atrioventricular canal, a malformation typically associated with DS, but the existence of a right aberrant azygos vein was not diagnosed until the postmortem study was performed. In addition, the study could not confirm the observed diverticulum of Kommerel in the origin of the left aberrant subclavian artery. This is likely because at this early gestational age, the saccular cavity was so tiny that it collapsed when the intravascular pressure disappeared. Finally, microdelections of the chromosome 22 have been associated with anomalies of the aortic arch [10], and with DS [11], however, genetic tests for this condition turned out to be negative.

In summary, we describe one of the few cases of DS associated with RAA/ALSA and report for the first time an association between DS and dilated cavum septi pellucidi, amplifying the long list of DS-related conditions accessible to prenatal ultrasound.
